# Activated protein C ameliorates *Bacillus anthracis* lethal toxin-induced lethal pathogenesis in rats

**DOI:** 10.1186/1423-0127-19-98

**Published:** 2012-11-21

**Authors:** Jyh-Hwa Kau, Yung-Luen Shih, Te-Sheng Lien, Chin-Cheng Lee, Hsin-Hsien Huang, Hung-Chi Lin, Der-Shan Sun, Hsin-Hou Chang

**Affiliations:** 1 Institute of Preventive Medicine, National Defense Medical Center, Taipei, Taiwan, ROC; 2 Department of Pathology and Laboratory Medicine, Shin-Kong Wu-Ho-Su Memorial Hospital, Taipei, Taiwan, ROC; 3 School of Medical Laboratory Science and Biotechnology, Taipei Medical University, Taipei, Taiwan, ROC; 4 Department of Molecular Biology and Human Genetics, Tzu-Chi University, Hualien, Taiwan, ROC

**Keywords:** Anthrax, Lethal toxin, Activated protein C, Coagulopathy

## Abstract

**Background:**

Lethal toxin (LT) is a major virulence factor of *Bacillus anthracis*. Sprague Dawley rats manifest pronounced lung edema and shock after LT treatments, resulting in high mortality. The heart failure that is induced by LT has been suggested to be a principal mechanism of lung edema and mortality in rodents. Since LT-induced death occurs more rapidly in rats than in mice, suggesting that other mechanisms in addition to the heart dysfunction may be contributed to the fast progression of LT-induced pathogenesis in rats. Coagulopathy may contribute to circulatory failure and lung injury. However, the effect of LT on coagulation-induced lung dysfunction is unclear.

**Methods:**

To investigate the involvement of coagulopathy in LT-mediated pathogenesis, the mortality, lung histology and coagulant levels of LT-treated rats were examined. The effects of activated protein C (aPC) on LT-mediated pathogenesis were also evaluated.

**Results:**

Fibrin depositions were detected in the lungs of LT-treated rats, indicating that coagulation was activated. Increased levels of plasma D-dimer and thrombomodulin, and the ameliorative effect of aPC further suggested that the activation of coagulation-fibrinolysis pathways plays a role in LT-mediated pathogenesis in rats. Reduced mortality was associated with decreased plasma levels of D-dimer and thrombomodulin following aPC treatments in rats with LT-mediated pathogenesis.

**Conclusions:**

These findings suggest that the activation of coagulation in lung tissue contributes to mortality in LT-mediated pathogenesis in rats. In addition, anticoagulant aPC may help to develop a feasible therapeutic strategy.

## Background

Anthrax is a disease caused by infection with the Gram-positive bacterium *Bacillus anthracis*. Anthrax is characterized by massive bacteremia in the absence of an effective immune response. Lethal toxin (LT) is a major virulence factor for B. anthracis that plays vital role in pathogenesis and the suppression of the host immune response [1,2]. LT is a binary protein complex that is composed of protective antigen (PA), a host-cell receptor binding subunit that mediates the cellular entry of its LT-counterpart, the lethal factor (LF), which is a metalloprotease that inactivates the mitogen-activated protein kinase kinases (MEKs) signaling pathways [2]. Although LT treatments might not reflect all features of *B. anthracis* infections, reductionistic approaches investigating the effects of LT *in vitro* and *in vivo* have aided in the elucidation the underlying pathogenic mechanisms in anthrax [3,4]. LT treatments in rodents resulted in hemorrhage, shock, and hypoxic tissue damages with high mortality [5-10].

In contrast to sepsis, treatments of LT do not induce strong proinflammatory cytokine and nitric oxide releases in rodents [4,8,11]. LT-treated rats displayed acute lung edema and accelerated progression of pathogenesis, compared with mice [4]. Recent findings suggest that LT can suppress cardiac function [3,12-14]. Because acute heart dysfunction can cause the accumulation of body fluid in the lungs [15,16], the lung edema associated with LT treatments in rodents may be a secondary manifestation of the failing heart [3,4,7,14]. Since LT-induced death is much faster in rats than in mice, this suggests that other mechanisms in addition to the heart dysfunction may be contributed to the fast progression of LT-induced pathogenesis in rats. Coagulopathy causes hemodynamic changes [17,18], and the conversion of extravascular fibrinogen into fibrin in the airway lumen can exacerbate lung injury [19,20]. LT–induced coagulopathy has been observed in mice [5,6]. However, the role of coagulopathy in LT-treated rats remains largely unclear.

Featured by abnormal activation of coagulation system, coagulopathy is frequently observed in a severe pathological condition like sepsis, which manifests with prolonged plasma clotting time and decreased circulating anticoagulant protein C [21,22]. Protein C is an inactive zymogen of a soluble, vitamin K–dependent, plasma serine protease that plays a central role in endogenous anticoagulation [23]. The activation of protein C requires binding to the endothelial protein C receptor and the thrombomodulin-thrombin complexes on the endothelium [23]. Decreased circulating protein C and increased circulating thrombomodulin are indicators of dysfunctional anticoagulation that lead to the prothrombotic, hypercoagulable states which are associated with sepsis and acute lung injury [21,24-28]. *B. anthracis* infections in humans and animals may lead to coagulopathy with elevated levels of plasma D-dimer [29-31]. Released into the circulation during the degradation of fibrin during fibrinolysis, D-dimer is widely used as a marker for the detection of coagulopathy [32]. Anthrax LT is a virulence factor of *B. anthracis*. However, such coagulant pathological changes were primarily observed in *B. anthracis* infections but not clearly demonstrated in LT-treated rats.

We investigated the role of coagulopathy in LT-induced pathogenesis in rats. Analyses of tissue sections, plasma clotting time, key coagulant factors, and hemodynamic parameters were performed to evaluate the coagulant status of LT-treated rats. Our analysis of plasma D-dimer and thrombomodulin levels suggest that coagulopathy is involved in LT-induced pathogenesis, and our observations of the ameliorative effects of activated protein C (aPC) on LT-induced coagulopathy support our findings. The regulation of coagulation and the pathological responses in rats are compared with those of mouse models.

## Methods

### Animals

Male Sprague Dawley rats aged 8 to 10 weeks that were free from specific pathogens, were purchased from the National Laboratory Animal Center, Taiwan. Rats were intravenously treated with either 0.25 mg/kg of PA or 0.25 mg/kg of LT (LF : PA = 15 : 85; a lethal dose), respectively. Rats will die from LT-induced pathogenesis at 4 to 7 hours following the LT injections. Lipopolysaccharide (LPS, Sigma-Aldrich, St. Louis, MO, USA) was dissolved in phosphate-buffered saline (PBS, pH 7.4) and used as a control to induce coagulopathy. The LPS treatments (20 mg/kg) were performed over the same time course as PA and LF injections. Xigris® (drotrecogin alfa, Eli Lilly & Co., Giessen, Germany), a recombinant form of human aPC, was administrated intravenously every 4 hour (96 μg/kg/injection) for the total of three injections as a modification of the manufacturer’s instructions. Because the Xigris was diluted in normal saline, the control rats were injected with an equivalent amount of saline for comparison. The experiments were performed in accordance with institutional guidelines. All animal research methods were approved by the Animal Care and Use Committee of Tzu Chi University (approval ID: 97005).

### Toxin preparation and usage

Components of *Bacillus anthracis* lethal toxins, LF and PA, were prepared and purified as previously described [33-35]. Doses of LT contained LF and PA at a ratio of 15:85, respectively, such that 100 μg LT contained 15 μg LF plus 85 μg PA, which is roughly equivalent to the composition of native toxin. Purified proteins were analyzed by sodium dodecyl sulfate polyacrylamide gel electrophoresis (SDS-PAGE). Using Image J software (National Institutes of Health, Bethesda, MD, USA), 85-90% purity was estimated for the total protein fractions. LPS was monitored using a Limulus Amoebocyte Lysate QCL-1000 kit (Lonza, Walkersville, MD, USA), and the level of LPS contamination was less than 1 EU/mg LT, a level of LPS that we observed to be incapable of eliciting significant inflammatory cytokine IL-1 and TNF-α secretion *in vitro* and *in vivo* (data not shown).

### Analysis of plasma proteins in plasma and lung tissue fluid

We measured the relative levels of albumin, immunoglobulin G (IgG) and fibrinogen in the plasma/lung homogenate fluid of surviving rats at four hours following the LT treatments (0.25 mg/kg) using enzyme-linked immunosorbent assay (ELISA). This time point demonstrated 30% mortality and therefore was expected to reveal pathophysiological changes of the lethal pathogenesis. The lung tissue of rats (0.5 g) was homogenized in 1 mL extraction buffer (10 mM Tris, 150 mM NaCl, pH 7.4) at 4°C using a tissue homogenizer (PRO Scientific, Oxford, CT, USA). The homogenates were transferred to 1.5 mL Eppendorf tubes, and centrifuged at 13,000 × g for 10 minutes at 4°C. The supernatant (lung-tissue fluid) was stored at −80°C until analyzed. The total protein in the plasma and lung-tissue fluid was determined by the method of Bradford. Plasma or lung tissue fluid (50 μg/well) was coated on 96-well dishes with at 37°C for 2 hours. After blocking with 5% BSA for additional 1 hour and 3 washes, the wells were then separately probed using anti-albumin (Abcam, Cambridge, MA, USA), anti-rat IgG (Jackson Immunoresearch Laboratory, West Grove, PA, USA) and anti-fibrinogen (Abcam) antibodies. Plasma of phosphate-buffered saline (PBS, pH 7.4)-treated rats served as a vehicle control. Plasma samples were used neat or diluted four-fold with PBS to keep the ELISA signal in a linear range. The plasma protein levels of treated rats were normalized to those of the vehicle controls.

### Histology and immunohistochemistry

Lungs of animals treated with LT (0.25 mg/kg) for 4 hours were fixed with 10% buffered formalin (Sigma-Aldrich) and embedded in paraffin. Tissue sections were then deparaffinized and stained with hematoxylin and eosin (H&E), and immunoglobulin (Ig). Tissue section images were obtained using a Leica DMIRE2 microscope (Wetzlar, Germany). Staining of fibrin was performed as previously described [36] using deparaffinization; 3% H_2_O_2_, 10 minutes; Tris-buffered saline (TBS) with 0.05% Tween (TBST); 0.1% proteinase K in TBS, 10 minutes; TBST; 1:1000 chicken anti-human fibrinogen (Abcam, Cambridge, MA), which cross reacts with rat fibrinogen and fibrin (data not shown), in an overnight incubation at 4°C. Bound primary antibody was visualized using an anti-chicken antibody-peroxidase conjugate and the DAB (3,3’-diaminobenzidine tetrahydrochloride) substrate, and sections were counterstained with hematoxylin. Purified total IgGs from non-immunized rabbits were used for the control staining.

### Hemoglobin analyses of lung fluid

Whole blood samples were collected from the tail veins of surviving rats at 4 hours following LT treatment and mixed 9:1 with ACD anticoagulant solution (38 mM citric acid, 75mM sodium citrate, 100mM dextrose) [37,38]. These blood samples were used as controls for comparisons to lung tissue and interstitial fluid. The hemoglobin levels were determined using a Drabkin’s reagent (Sigma-Aldrich) (ACD and blood : Drabkin’s reagent = 1:500) according to previously described methods [39,40]. Serially dilutions were used to plot a standard curve. Hemoglobin levels in the samples from LT-treated rats were normalized to the levels in the whole blood of vehicle controls. The 1:4 dilutions of control blood were used as reference points to maintain a linear data range within the detection limits of the method.

### Blood oxygen measurement

At 4 hours following the LT treatments, surviving rats were anaesthetized by intraperitoneal injection of pentobarbital (30 mg/kg). Cardiac puncture of the (right ventricle) was used to collect blood in 50 IU/ml lithium heparinized syringes, and the blood oxygen content (PO_2_) was measured directly by the method of amperometry in a blood gas analysis machine (Synthesis series, Instrumentation Laboratory, Lexington, MA, USA).

### Cell death analysis

The mouse macrophage-like cell line J774A.1 (*ATCC* TIB67) was used for cytotoxicity analysis [34,35,41]. The cells were maintained in Dulbeco’s modified Eagle’s medium (DMEM) containing 10% fetal bovine serum (FBS). The cells (1 × 10^5^/well) were treated with a cytotoxic dose of LT (15 ng/mL), with or without additional treatments of aPC (Xigris; 1.5 μg/mL), in 96-well dishes for 3 hours. The level of viable cells was determined using the WST-1 kit (Roche Diagnostics, Indianapolis, IN, USA) according to the manufacturer’s instructions.

### Coagulant analyses

Plasma levels of D-dimer, thrombomodulin, protein C and antithrombin III levels in mice were determined before (0 hour), and after (2 and 4 hours) PA, LT and LPS treatments using a D-dimer ELISA kit (American Diagnostica, Stamford, CT, USA), a thrombomodulin ELISA kit (Abcam) and chromogenic protein C and antithrombin III kits (American Diagnostica), respectively [36]. Activated partial thromboplastin time (aPTT) and prothrombin time (PT) analyses were performed to distinguish between the effects of LT on the intrinsic and extrinsic coagulation pathways, using an ACL Futura Plus coagulometer (Instrumentation Laboratory) as described [5].

### Statistical analysis

Means and standard deviations for quantifiable data were calculated using Microsoft Office Excel 2003 for Windows. Comparisons between groups of nonparametric data were made using an ANOVA. For survival studies, the log-rank test was performed to determine significance using the Online Application Survival Analysis Lifespan Assays (http://sbi.postech.ac.kr/oasis) [42]. The unpaired Student’s *t* test was used to analyze the parametric data for other results. Results with *p* values less than 0.05 were considered statistically significant.

## Results

### Pulmonary abnormalities

**Figure 1:**
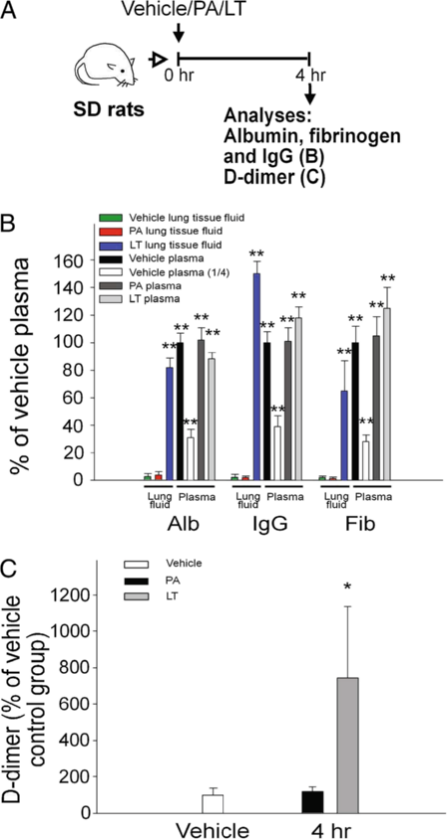
**Analyses of lung fluid and coagulant abnormalities.** Sprague Dawley rats were treated with 0.25 mg/kg protective antigen (PA) or 0.25 mg/kg lethal toxin (LT) for 4 h to induce pathogenesis. The experimental outline is illustrated in panel **A**. (**B**) Relative levels of albumin (Alb), immunoglobulin (IgG), and fibrinogen (Fib) in rat plasma and lung homogenate tissue fluid were determined. Protein levels in plasma of the vehicle control group were adjusted to 100% in the respective groups (Alb: 3.24 ± 0.26 g/dL, IgG: 797 ± 86 mg/dL, Fib: 119 ± 8 mg/dL; B, vehicle groups, *n* = 5; ** *p* < 0.01 compared to lung tissue fluid of both vehicle and PA groups). (**C**) Relative plasma D-dimer levels were also determined (*n* = 5; * *p* < 0.05, LT-4hr vs. PA-4 h groups). (**B**-**C**) Data were obtained from two experiments with 2 and 3 replicates, respectively.

**Figure 2:**
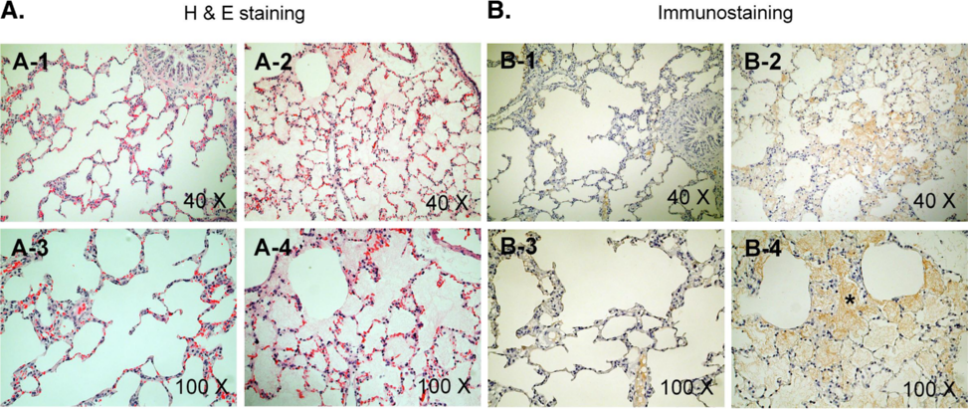
**Lung tissue sections of rats.** Protective antigen (PA) and lethal toxin (LT) (0.25 mg/kg) were injected into Sprague Dawley rats for four hours to induce pathogenic responses. Lung tissue sections from the PA- (A-1,3, B-1,3) and the LT- (A-2,4, B-2,4) treated rats were stained with hematoxylin and eosin (**A**) and anti-fibrinogen/fibrin antibodies (**B**). The asterisks * refer to the brown-colored regions that indicate the localization of the anti-fibrinogen/fibrin antibodies (B-4).

**Figure 3:**
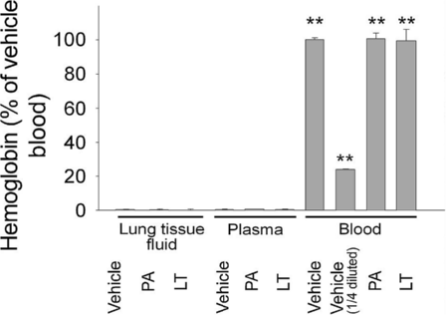
**Hemoglobin analysis.** Hemoglobin levels in lung tissue homogenate fluid, plasma, and whole blood from PA- and LT-treated rats were determined. The hemoglobin level of whole blood from the vehicle group was set at 100% for the normalization of the data. The vehicle (1/4 diluted) data indicate the reference point of whole blood samples of vehicle-treated group that were diluted to 25% by the addition of phosphate buffered saline. Data were obtained from 3 experiments with 2 replicates (34*n* = 6). ** *p* < 0.001, compared to the vehicle lung tissue fluid group.

**Figure 4:**
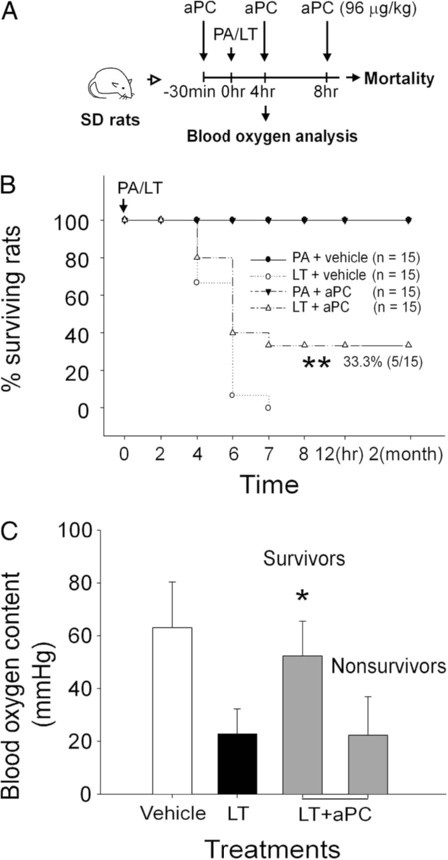
**Activated protein C (aPC) treatments reduce lethal toxin (LT)-mediated mortality.** (**A**) The experimental outline is illustrated. (**B**) The comparison of the mortality of LT-treated rats with or without aPC at 0 to 12 h following protective antigen (PA)- or LT-treatments (LT, 0.25 mg/kg single dose; aPC, 96 μg/kg/4 h; *n* = 15) was plotted as Kaplan-Meier curves (LT vs. LT + aPC, ** *p* < 0.005). The survivors in the LT + aPC group were observed for 2 mo. (**C**) The blood oxygen content of rats after LT (0.25 mg/kg) and LT plus aPC (LT + aPC) treatments for 4 h. Vehicle and LT data were collected from 3 experiments with 2 replicates (*n* = 6). The data for LT + aPC survivors and nonsurvivors were collected from 2 experiments with 2 replicates (*n* = 4; * *p* < 0.05 compared to the LT group). The nonsurvivors were those rats that survived the first 4 h of treatment, but died later (4 to 7 h). The survivors were those rats that survived the entire course of treatment (B and C; LT + aPC groups).

Sprague Dawley rats were intravenously treated with a lethal dose of LT (0.25 mg/kg, LF : PA = 15 : 85, a ratio approximately equivalent to *Bacillus anthracis* derived-LT; 100% mortality) to investigate LT-induced pathogenesis. Mortality occurred approximately 4 to 7 hours after the treatment. One of the most prominent pathological signs in LT-treated rats is the lung edema [7,43]. To verify whether extravascular plasma or secreted-fluid from lung cells had contributed to the lung edema, the levels of albumin, IgG and fibrinogen in the lung homogenate fluid were examined. The ELISA data revealed that these plasma proteins were all detectable in the lung tissue fluid following LT-treatments. The levels of the plasma proteins in the lung fluid were approximately equivalent to those in the plasma of vehicle controls, and were significantly higher than those of lung tissue fluid of both the vehicle controls and the PA-treatments (Figure 1A, experiment outline, 1B, ** *p* < 0.01, vs. lung tissue fluid of vehicle and PA groups). These results suggest that the lungs of LT-treated rats experienced massive extravascular plasma leakage. D-dimer is a fibrinogen protein fragment produced upon a coagulation-fibrinolytic activation. D-dimer was significantly elevated in the plasma following LT treatments (Figure 1C), compared with PA control rats (*p* < 0.05), which indicates that the coagulation-fibrinolytic cascade was initiated. H&E staining (Figure 2A) and immunohistochemistry (Figure 2B) using anti-fibrinogen/fibrin antibodies were performed to examine the coagulant activation within the alveoli, and severe lung edema was observed, which is consistent with previous reports [7,43]. The H&E staining pattern of lung tissue sections of PA-treated rats was similar to the vehicle control groups (data not shown). Thus, PA did not induce pathological responses in rats. Compared to the PA groups (Figure 2A-1,3), the alveoli of LT-treated rats contained significantly more fluid (Figure 2A-2,4). Immunohistochemistry data also showed that extravascular fibrinogen and fibrin were distributed throughout the fluid-containing alveoli of LT-treated rats, but were rare in PA-treated controls (Figure 2, fibrinogen/fibrin: brown-color stained regions, estimate to occupy approximately 50% of alveoli of LT groups; PA: 2B-1,3, vs. LT: 2B-2,4). The strong immunostaining of fibrinogen/fibrin was observed in the lung tissue of LT-treated animals (Figure 2B-4, asterisk marked brown-color fibrous substances). These results suggested that coagulant cascades were initiated in the lungs of LT-treated rats.

To determine whether the LT-induced lung edema was caused by increased vascular permeability or by hemorrhage, the hemoglobin content of lung tissue fluid was compared with that of plasma and whole-blood samples. Only background levels of hemoglobin were detected in the lung tissue fluid of LT-treated rats (Figure 3, LT lung tissue fluid vs. vehicle, PA and LT plasma groups). Despite previous reports that LT induced hemolysis *in vitro*[44], LT treatment did not cause significant hemolysis of erythrocytes in our *in vivo* experiments, compared with control and PA-treated rats (Figure 3). Thus, the LT-induced lung edema was likely caused by extravascular plasma (Figure 1B), rather than hemorrhaging (Figure 3, no induction of hemoglobin in LT lung tissue fluid groups). Although, systemic coagulopathy is frequently accompanied by thrombocytopenia [22], no systemic changes in platelet counts have been observed in LT-treated rats [11]. Consistent with the results of previous studies [11,45], the complete blood count data showed that the PA and LT treatments did not significantly reduced the levels of platelets, erythrocytes, or leukocytes in LT-treated rats (data not shown). In addition, our examination of the brain, heart, lung, liver, kidney, spleen, and intestine of LT-treated rats showed that only the lung tissues exhibited significant extravascular fibrin staining, compared with PA-treated rats (Figure 2, lung; other data not shown). Thus our D-dimer and immunohistochemistry data (Figure 1 and 2) suggest that the activation of the coagulation-fibrinolysis cascades was restricted to the lung.

### Ameliorative effect of activated protein C in LT-mediated pathogenesis

Fibrin inactivate surfactants, and coagulation activation in the lung has been shown to exacerbate allergic airway inflammation [20]. In contrast, anticoagulants have been shown to have an ameliorative effect on lung inflammation [19,46]. Therefore, we evaluated the effect of recombinant aPC on LT-induced pathogenesis in rats. The data revealed that aPC treatments significantly reduced LT-mediated mortality, compared with PA-treated controls (Figure 4A, experiment outline; 4B, LT 0% survival, vs. LT + aPC 33% survival; LT vs. LT + aPC, ** *p* < 0.005). In addition, aPC-mediated amelioration on blood oxygen levels were also correlated to the mouse survival (Figure 4C, amelioration in LT + aPC treated survivors, but not nonsurvivors; * *p* < 0.05, vs. LT groups). To determine whether the protective effect of aPC was the result of the direct inactivation of LT through protease activity of aPC, cytotoxicity experiments were performed using mouse macrophage J774A.1 cells, as described [34,35]. The results of the cytotoxicity experiments showed that aPC treatments did not significantly rescue the J774A.1 cells from the effects of the LT-mediated cytotoxicity (data not shown), indicating that aPC does not inactive LT through a direct inactivation.

**Figure 5:**
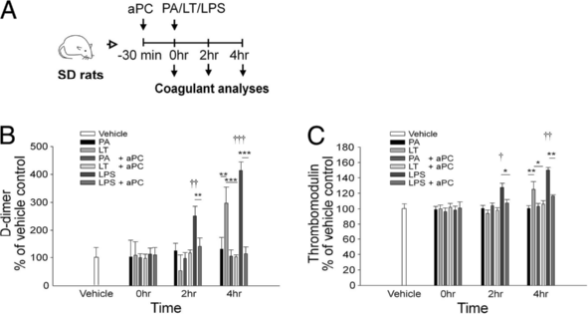
**Analysis of the effects of activated protein C (aPC) in rats treated with protective antigen (PA), lethal toxin (LT), or lipopolysaccharide (LPS).** The experimental outline is illustrated in panel **A**. The plasma D-dimer (**B**) and thrombomodulin (**C**) levels of PA-, LT-, or LPS-treated rats with and without aPC treatment at 0, 2, and 4 h following the PA, LT, or LPS treatments. The LPS treatments served as positive controls for the induction of coagulopathy (B-C, LPS groups: induction of D-dimer, thrombomodulin in B, C). In panels B and C, the levels for the vehicle group were set at 100 % for the normalization of the data. For the comparisons that are indicated in B and C, * *p* < 0.05, ** *p* < 0.01, and *** *p* < 0.001. For the comparisons to the vehicle group, † *p* < 0.05, †† *p* < 0.01, ††† *p* < 0.001. Data were collect from 3 experiments with 2 replicates (*n* = 6).

Induction of plasma D-dimer and thrombomodulin are associated with coagulopathy and lung injury [27,32,36]. To investigate whether the ameliorative effect of aPC is associated with the suppression of coagulopathy, the plasma levels of D-dimer and thrombomodulin were compared to other coagulant parameters including aPTT, PT, protein C and antithrombin III. Because aPC has an ameliorative effect on LPS-induced coagulopathy [21,22], the amelioration of LT-induced coagulopathy was compare to that caused by LPS-treatments (20 mg/kg), and the results showed that aPC treatments significantly suppressed both the LT- and the LPS-mediated induction of plasma D-dimer and thrombomodulin (Figure 5A, experiment outline; 5B, D-dimer; 5C, thrombomodulin). However, the data from those survivors may tend to show a relatively weaker effect (Figure 5, 1-h groups) because mortality was first observed at 4-h following LT treatment (Figure 4B). Therefore, the extent of the changes in LT-induced coagulopathy may not be fully demonstrated in our results (Figure 5). Systemic consumptive coagulopathy in sepsis may exhaust both coagulant and anticoagulant factors, which may be manifested as prolonged plasma clotting time [21]. The results of analyses of aPTT and PT showed that LT treatments did not significantly change the clotting time at 2 or 4 h following LT treatment, compared with vehicle controls (Additional file 1: Figure S1A and S1B). This is distinct from the effects of LPS treatment, which significantly increased clotting time (Additional file 1: Figure S1A and S1B, LPS vs. vehicle groups, † *p* < 0.05). Similarly, plasma levels of protein C and antithrombin III were significantly reduced by LPS treatment (*p* < 0.05), but were not reduced by LT treatments (Additional file 1: Figure S1C and S1D), and treatment with aPC ameliorated the LPS-induced reduction of protein C and antithrombin III (Additional file 1: Figure S1A and S1D, LPS vs. LPS + aPC groups, * *p* < 0.05, ** *p* < 0.01). These results suggest that systemic coagulopathy is not involved in LT-mediated pathogenesis in rats.

## Discussion

Pulmonary edema and lesions are frequently observed in inhalation anthrax [29,47,48]. Elevated plasma D-dimer indicates coagulopathy in anthrax patients [29,30]. These manifestations are reflected in the results of in the rat experiments described in this study. Compared to the rat model, several pathological features of coagulant regulation are distinct in mice. LT induces thrombocytopenia in mice [5,8], but not in rats [11]. The results of our D-dimer analyses suggest that coagulopathy is involved in LT-mediated pathogenesis in rats (Figure 5B), which is consistent with anthrax in human [29,30], but not in mice [5]. The protective role of aPC in LT-mediated mortality in rats (Figure 4B), further suggests a role of coagulopathy. In contrast, the lack aPC-mediated amelioration of LT-induced pathogenesis in the C57Bl/6J mice in our lethal dose experiments indicates that coagulopathy does not contribute significantly to mortality in mice (data not shown). Despite these distinct differences in coagulopathy, LT-induced cardiopathy is similar in the rat and mouse models [3,4,7,12-14]. The significant differences in lung pathology indicate a mechanism other than heart dysfunction. Thus, coagulopathy was a consideration. Although no obvious systemic changes in clotting time or plasma anticoagulants were observed, fibrin deposition was observed in the lung sections, suggesting that the lung is the primarily organ affected by LT-induced coagulopathy in rats.

In the absence of systemic coagulopathy, localized activation of coagulation in the lung may cause significant pathology. LT-induced pathogenesis in rats shares common features with acute lung injury and acute respiratory distress syndrome in humans, including multiple organ dysfunction, intra-alveolar coagulation with fibrin deposition on the hyaline membrane [49,50], and decreased mortality with aPC treatment [19]. Coagulant activation and the presence of fibrin is known to be associated with the pathophysiology of lung injury [19,20,51,52], and LT has been shown to increase the paracellular permeability of endothelial cells [53]. Our data indicate that this increased permeability results in the extravasation of fibrinogen and fibrin into the alveolar lumen (Figure 1B, 2L).

Fibrinogen, fibrin and related degradation products have been shown to suppress surfactant functions in the alveoli [51,52]. The activation of coagulation pathways can induce local leukocyte traffic and endothelial cell permeability [54] that may exacerbate lung injury [20,54]. The elevation of D-dimer following LT treatment indicates the initiation of the coagulation-fibrinolysis cascade (Figure 1C; Figure 5B). Unlike endothelial cells, the cells comprising the alveolar surface do not express thrombomodulin. The LT-induced release of endothelial thrombomodulin into the plasma that supposedly will suppress vascular anticoagulant protein C pathway. Because the LT-induced coagulopathy is primarily restricted to the lung, the circulating plasma level of aPC may not increase in the same manner as occurs in LPS-treated animals (Figure 5F, LT vs. LPS). Since aPC can inactivate the coagulation cascade and block fibrin formation [23], this suggests the ameliorative role of aPC in LT-treated animals (Figure 4f, 5). The anticoagulant and anti-inflammatory activities of aPC play protective roles in lung injury and asthma [55]. Because LT-mediated pathogenesis does not induce significant secretion of proinflammatory cytokines [8,11], anticoagulant activity of aPC is likely the major effect that contributes to the amelioration of LT-induced pathogenesis.

Recombinant aPC (Xigris) was previously approved by United States Food and Drug Administration (FDA) for the reduction of mortality in adult patients with severe sepsis [56]. Xigris was, however, withdrawn from the market by the manufacturer after the failure of its world wide trail to treat severe sepsis [57,58]. LT-mediated pathogenesis does not induce a prominent inflammatory response [8,11], and the changes in aPTT, PT, protein C, and antithrombin III are distinct from those of endotoxin-induced sepsis (Figure 5D and 5G). Thus, LT-induced pathogenesis is significantly different from sepsis. As a result, Xigris may, nonetheless, ameliorate LT-mediated pathogenesis by re-establishing the regulation of coagulation. Further investigations of the ameliorative effects of Xigris on LT-mediated pathogenesis are warranted.

Consistent with the results of previous studies [12,13], the mean blood pressure of the LT-treated rats significantly decreased at approximately 4 h following LT treatment (Additional file 1: Figure S2A vs. Figure S2B). To ascertain whether the elicitation of lung edema occurred prior to the circulatory collapse, we examined the extent of lung injury by measuring the blood pressure and the lung wet-to-dry weight ratio of LT-treated rats. We found that both pulmonary and circulatory abnormalities were induced over a similar time course (Additional file 1: Figure S2A-B; 4-hour, LT + vehicle vs. vehicle groups). The changes in levels of plasma D-dimer and thrombomodulin were also associated with a similar time course (Figure 5B-C, 4-hour groups). Thus, the LT treatments elicited both coagulation-mediated lung injury (Figure 5 and Additional file 1: Figure S2C) and heart dysfunction (Additional file 1: Figure S2B) in rats. Therefore, we propose a hypothetical model for LT-mediated pathogenesis that is based on the hemodynamic changes and the altered coagulation occurring simultaneously (Additional file 1: Figure S3).

Severe heart failure leads to the redistribution and accumulation of body fluid in the lung [15,16], which would likely exacerbate the increased vascular permeability and the coagulation-activation in the lungs of LT-treated rats. Coagulopathy may also negatively influence cardiac function, resulting in further hemodynamic changes [17,18]. Dysregulation of protein C activation and thrombosis are also caused by secondary pulmonary hypertension, a known effect of severe heart failure [59]. As a result, these two pathogenic events may have potential for mutual exacerbation (Additional file 1: Figure S3). Thus, the interruption of this exacerbating feedback by aPC treatment may contribute to the improved survival rate (Additional file 1: Figure S3). The cross-talks between these two types of pathogenic regulations are interesting issues and worthy to be further investigated.

In addition to LT, *B. anthracis* releases other toxins that perturb coagulation and vascular processes during infection. Bacteria-derived metalloproteases have been shown to degrade von Willebrand factor and ADAMTS13, which contribute to the recruitment of platelets to the injured vessel wall [60]. In addition, bacterial protease InhA has been shown to inhibit fibrinolysis by activating plasminogen activator inhibitor-1 [61-63], and anthrolysin O, a cholesterol-dependent cytolysin and a Toll-like receptor 4 agonist, has been shown to disrupt endothelial and epithelial barriers [64-66]. Combined treatments of anthrolysin O and edema toxin significantly induced thrombin activity in human umbilical vein endothelial cells [67]. These studies indicate that *B. anthracis*-derived secretory factors play important roles in anthrax consumptive coagulopathy. Further investigations are needed to reveal the combined effects of these virulence factors on circulatory homeostasis during infection.

## Conclusions

In summary, our results suggest that the activation of the coagulation-fibrinolysis cascade is involved in LT-mediated pathogenesis in rats. The anticoagulant aPC significantly reduced mortality in LT-treated rats. Specific treatments to overcome LT-mediated pathogenesis are lacking. Our findings may aid in the development of new therapeutic strategies for treating anthrax infections.

## Competing interests

The authors declare no potential conflict of interests.

## Authors’ contributions

JHK, DSS, HHC designed the experiments. JHK, YLS, TSL, CCL carried out the cytotoxicity, tissue section, blood and plasma protein analyses, coagulant parameter analyses, mortality and aPC-rescue experiments. HHH, HCL provided anthrax lethal toxin. HHC drafted the manuscript. All authors read and approved the final manuscript.

## Supplementary Material

Additional file 1 Coagulant, lung edema and blood pressure analyses, and a hypothetical model. Click here for file
