# Supplementary material for: Activated protein C ameliorates Bacillus anthracis lethal toxin-induced lethal pathogenesis in rats
Source: J Biomed Sci. 2012 Nov 21;19(1):98. doi: 10.1186/1423-0127-19-98 (PMC3536616; doi:10.1186/1423-0127-19-98)
Supplement: Additional file 1 — Coagulant, lung edema and blood pressure analyses, and a hypothetical model. [file 1423-0127-19-98-S1.doc]

**SUPPLEMENTAL MATERIALS**

**Coagulant, lung edema and blood pressure analyses, and a hypothetical model.**

**I. Supplemental methods**

**Lung weight ratios and blood pressure.** The extent of lung edema was measured by wet-to-dry tissue weight ratios using previously described methods . After treated with LT or PA for various time points, rats were sacrificed. After dissection, fresh lung was weighted. Lungs were then placed in a drying oven for 24 hour at 80°C until a constant weight was obtained. Systemic blood pressure was determined by the tail cuff method using the BP 2000 analysis system (Visitech Systems) .

**II. Supplemental figures**

**Figure S1. Coagulant analyses of PA/LT and aPC treated rats.**

The plasma clotting time aPTT (A) and PT (B), and the plasma level of anticoagulants protein C (C) and antithrombin III (ATIII) (D) of rats treated with or without PA/LT and aPC at time courses 0, 2 and 4 hour post PA/LT treatments were shown. The LPS treatments were served as positive controls on the elicitation of coagulopathy (A-D, LPS groups: prolonged clotting time in A-B, suppressed anticoagulants in C-D). In panels C and D, the levels of vehicle groups were normalized to 100 %. * *p* < 0.05, ** *p* < 0.01, compared with LPS + aPC groups in A-D. † *p* < 0.05, compared to vehicle groups. ****ND in LPS 4-hour groups: no detectable clotting (B). n = 6, three experiments with 2 replicates.

**
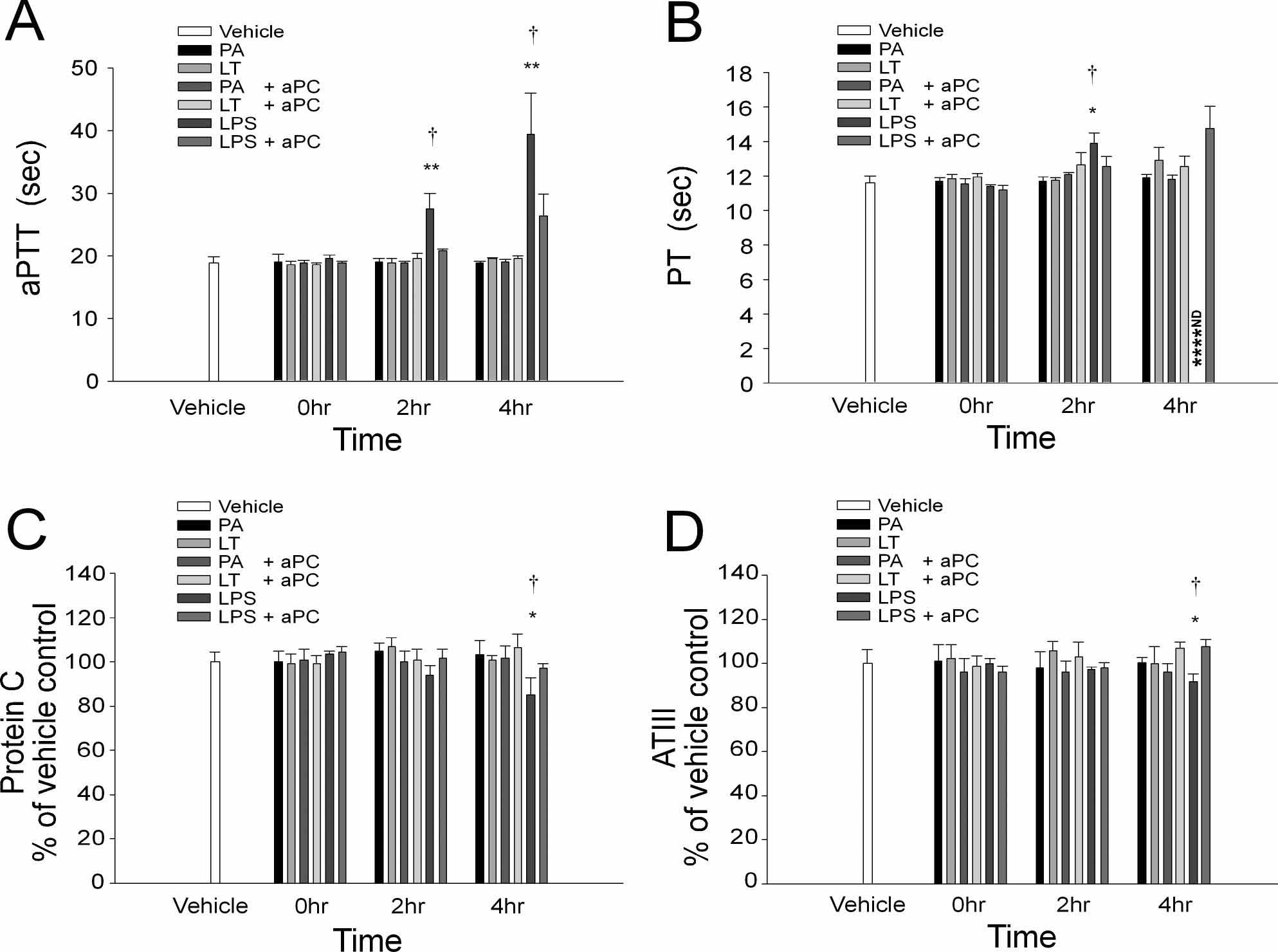
Figure S1**

**Figure S2. Time courses of lung edema versus blood pressure.**

Effect of LT and PA treatments on the changes in wet-dry-dry weight ratio (A) and systemic blood pressure (B) at various time points were shown. Mean arterial blood pressure of untreated groups was normalized to 100 %. * *p* < 0.05, ** *p* < 0.01.

**
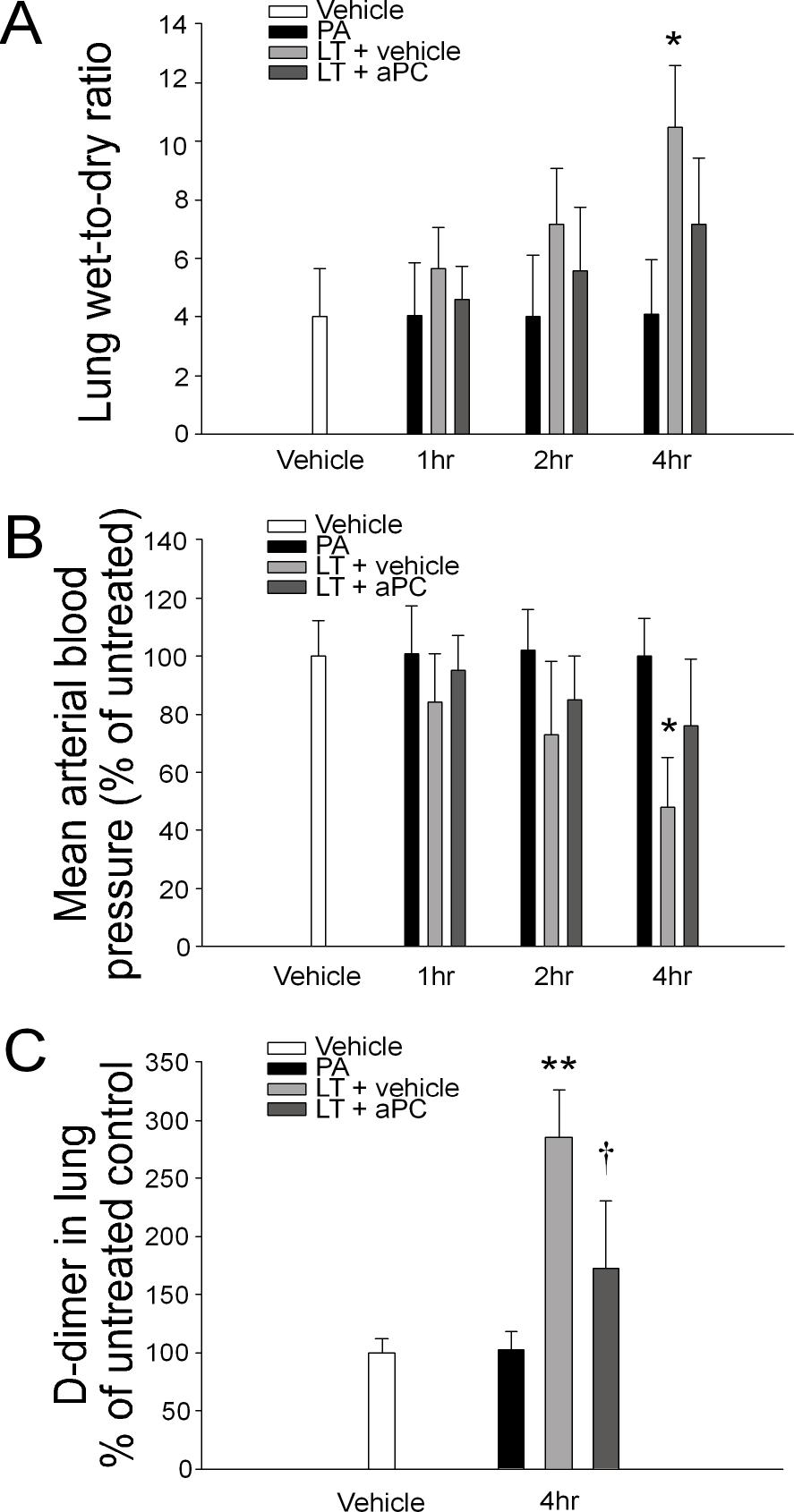
**

**Figure S2**

**Figure S3. Hypothetical model.**

LT treatments elicit coagulant and hemodynamic changes in rats. Both pathological events likely contribute to the mortality. These two pathological events might exacerbate each other and eventually lead to mortality (question mark: the potential pathways); while such pathological alterations could be ameliorated by aPC treatments. ↓ Activation processes; ⊥ Inhibition processes.


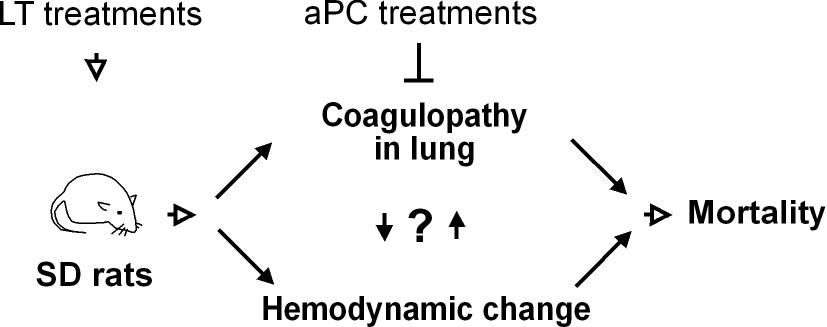


**Figure S3**

**III. Supplemental references**

1. Yildiz G, Demiryurek AT, Gumusel B, Lippton H: **Ischemic preconditioning modulates ischemia-reperfusion injury in the rat lung: role of adenosine receptors.** *Eur J Pharmacol* 2007, **556:**144-150.

2. Hansmann G, de Jesus Perez VA, Alastalo TP, Alvira CM, Guignabert C, Bekker JM, Schellong S, Urashima T, Wang L, Morrell NW, Rabinovitch M: **An antiproliferative BMP-2/PPARgamma/apoE axis in human and murine SMCs and its role in pulmonary hypertension.** *J Clin Invest* 2008, **118:**1846-1857.
